# Appropriate coronary revascularization can be accomplished if myocardial perfusion is quantified by positron emission tomography prior to treatment decision

**DOI:** 10.1007/s12350-019-01938-y

**Published:** 2019-11-08

**Authors:** Shahnaz Akil, Fredrik Hedeer, Jenny Oddstig, Thomas Olsson, Jonas Jögi, David Erlinge, Marcus Carlsson, Håkan Arheden, Cecilia Hindorf, Henrik Engblom

**Affiliations:** 1 Department of Radiological Sciences, College of Health and Rehabilitation Sciences, Princess Nourah Bint Abdulrahman University, Riyadh, Saudi Arabia; 2 Department of Clinical Sciences Lund, Clinical Physiology, Skane University Hospital, Lund University, Lund, Sweden; 3 Radiation Physics, Skane University Hospital, Lund, Sweden; 4 Department of Clinical Sciences, Cardiology, Skane University Hospital, Lund University, Lund, Sweden

**Keywords:** Coronary artery disease, coronary angiography, revascularization, stress imaging

## Abstract

**Background:**

Many patients undergo percutaneous coronary intervention (PCI) without the use of non-invasive stress testing prior to treatment. The aim of this study was to determine the potential added value of guiding revascularization by quantitative assessment of myocardial perfusion prior to intervention.

**Methods and Results:**

Thirty-three patients (10 females) with suspected or established CAD who had been referred for a clinical coronary angiography (CA) with possibility for PCI were included. Adenosine stress and rest ^13^N-NH_3_ PET, cardiac magnetic resonance (CMR), and cardiopulmonary exercise test were performed 4 ± 3 weeks before and 5 ± 1 months after CA. The angiographer was blinded to the PET and CMR results. Myocardial flow reserve (MFR) < 2.0 by PET was considered abnormal. A PCI was performed in 19/33 patients. In 41% (11/27) of the revascularized vessel territories, a normal regional MFR was found prior to the PCI and no improvement in MFR was found at follow-up (*P *= 0.9). However, vessel territories with regional MFR < 2.0 at baseline improved significantly after PCI (*P *= 0.003). Of the 14 patients not undergoing PCI, four had MFR < 2.0 in one or more coronary territories.

**Conclusion:**

Assessment of quantitative myocardial perfusion prior to revascularization could lead to more appropriate use of CA when managing patients with stable CAD.

**Electronic supplementary material:**

The online version of this article (10.1007/s12350-019-01938-y) contains supplementary material which is available to authorized users.

## Introduction

Stable coronary artery disease (CAD), characterized by the presence of atherosclerotic plaques in one or more of the main coronary arteries, is one of the leading causes of heart failure and cardiovascular death in the western world.^1^ If the plaques become stenotic it may cause stress-induced myocardial ischemia within the myocardium supplied by the affected artery.

Revascularization, either by the way of percutaneous coronary intervention (PCI) or a coronary artery bypass surgery (CABG), is aimed to treat coronary stenosis causing stress-induced ischemia 1 and thereby relieve symptoms as well as improve prognosis in patients with stable CAD.^2^ Therefore, successful elective revascularization is expected to restore the myocardial perfusion (MP) and the regional left ventricular function as well as improve the patient’s exercise capacity. It has been shown that coronary angiography (CA)-guided revascularizations have varying success 3-8 because the association between degree of coronary stenosis and presence of stress-induced ischemia is weak.^9^ Therefore, current guidelines recommend assessment of stress-induced ischemia non-invasively for accurate revascularization in patients with CAD.^10^ In current clinical practice following the NICE guidelines,^11^ however, elective revascularizations are often performed based on clinical history, risk factors, and invasive CA findings, without prior assessment of stress-induced ischemia 12,13 resulting in normal findings in many patients undergoing invasive CA.^14^ These unnecessary CA could potentially be reduced by non-invasive imaging, without any negative effect on major adverse cardiac events.^14^,^15^

Myocardial perfusion (MP) and myocardial flow reserve (MFR) can be quantified using cardiac positron emission tomography (PET) performed during adenosine stress and at rest, enabling non-invasive assessment of stress-induced myocardial ischemia.

The aim of this prospective study was to determine the potential added value of quantitative assessment of myocardial perfusion prior to intervention, for guidance of revascularization decision-making.

## Methods

### Study Population and Design

This prospective, blinded study initially included 37 (11 females, mean age 68 ± 7 years, age range 50 to 86 years) patients with suspected stable CAD, clinically referred for CA from November 2013 to April 2017. Patients with atrial fibrillation, claustrophobia, asthma, severe chronic obstructive lung disease, and glomerular filtration rate < 30 mL·min^−1^ were excluded.

All patients performed adenosine stress/rest ^13^N-NH_3_ cardiac PET and CMR on the same day (4 to 5 hours apart), followed by a cardiopulmonary exercise testing the day after to evaluate the myocardial perfusion, regional and global left ventricular function, presence of myocardial infarction, and peak oxygen uptake (VO_2_ peak). These baseline examinations were performed 4 ± 3 weeks before undergoing CA with or without percutaneous coronary intervention (PCI). All examinations were repeated after a follow-up period of 5 ± 1 months after the CA. Of the included 37 patients, four did not want to participate in the follow-up part of the study. Thus, a total of 33 patients with both baseline and follow-up were finally included in the study. Furthermore, follow-up cardiopulmonary exercise testing was not performed in 2/33 patients. 

By study design, the referring physician and the angiographer were blinded to the results from all baseline examinations (cardiac PET, CMR, and cardiopulmonary exercise test). Changes in medication after CA, with or without revascularization, were determined by the physician responsible for the patient, without any knowledge of the results from the cardiac PET, CMR, and cardiopulmonary exercise testing examinations. The study was approved by the Regional Ethical Committee, Lund, Sweden and written informed consent was obtained from all patients.

### Coronary Angiography

The CA was performed according to the national protocol used in clinical routine, and invasive flow reserve measurements were performed when deemed clinically indicated. Instant flow reserve (iFR) > 0.89 and fractional flow reserve (FFR) > 0.80 were considered normal, as previously suggested.^16^,^17^ The decision to perform a revascularization in conjunction with the coronary angiography was based on the available clinical data and coronary angiography findings in each of the three vessels (LAD, RCA, and LCX), according to clinical routine.

### PET Imaging

All patients performed the rest imaging first followed by stress imaging approximately 1 hour later. For the stress examination, ^13^N-NH_3_ was injected after 3 minutes of adenosine infusion (140 μg·kg^−1^·min^−1^). Adenosine infusion continued, with continuous ECG monitoring, for another 4 minutes after isotope injection. The injected activity of ^13^N-NH_3_ was 525 ± 78 MBq at both rest and stress.

For image positioning, a scout view over the chest was performed followed by a low-dose computed tomography for attenuation correction (120 kV; 10 mAs, 10; rotation time 0.5 seconds). The PET acquisition was started simultaneously with the isotope injection, for both rest and stress examinations. Images were acquired using a GE Discovery 690 PET/CT.

Before reconstructing the images, evidence for patient motion was checked between the CT and PET images and manual adjustments were made. The first 4 min of the PET acquisition were used to reconstruct the rest and stress dynamic images into 15 time frames (12 × 10 seconds, 2 × 30 seconds and 1 × 60 seconds) using OSEM (3 iterations, 12 subsets) and a 5 mm post-filter.

### PET Image Analysis

The reconstructed dynamic images were analyzed using the software Carimas (version 2.7, Turku, Finland). The left ventricle was delineated automatically with manual adjustments when needed. The activity in the blood (arterial input) and the myocardial wall as a function of time served as input data for the deGrado compartment model calculations 18 for ^13^N-NH_3._ Based on this input data, quantification of the stress and rest global MP in mL·min^−1^·g^−1^ as well as the regional MP in each of the three vessel territories (LAD, LCX, and RCA) was accomplished. The MFR was calculated by dividing the stress by rest MP. MFR< 2.0 was considered pathologic, as previously suggested.^19^,^20^

### CMR Imaging and Analysis

Patients were examined on either a 1.5 T Philips Achieva (Best, The Netherlands; *n* = 10) or Siemens Aera (Erlangen, Germany; *n *= 23), as the CMR scanner was replaced during the study. Cine steady-state free precession (SSFP) short-axis images were acquired covering the entire left ventricle (slice thickness 8 mm, no gap). Cine SSFP images were also acquired in the three standard long-axis planes (2-, 3-, and 4-chamber views). Approximately 15 minutes after an intravenous injection of 0.2 mmol·kg^−1^ gadolinium-based contrast agent (Dotarem, Gothia Medical, Billdal, Sweden), late gadolinium-enhanced (LGE) images were acquired in the corresponding image planes as for the SSFP cine images.

All CMR analysis was performed using the software Segment version 2.0 (http://segment.heiberg.se).^21^ Left ventricular ejection fraction (LVEF) and regional wall thickening were calculated after manual delineation of the endocardial and epicardial borders in end-diastole (ED) and end-systole (ES) in the cine short-axis images covering the entire left ventricle. The ED and ES regional wall thickness was determined and the regional wall thickening in % was calculated using the following formula: (ES wall thickness—ED wall thickness)/ED wall thickness, for each of the three vessel territories (LAD, LCX, and RCA) according to the 17-segment model.^22^ Thus, the most basal short-axis slices with myocardium not present throughout the circumference were excluded.

Presence of infarction and scar size was determined from the LGE short-axis images using a recently described automated EWA algorithm for scar quantification taking signal intensity distribution, coronary vessel territory, and partial volume effects into consideration.^23^

### Cardiopulmonary Exercise Testing

Bicycle exercise stress test with simultaneous gas analysis was performed according to clinical routine. After acquiring an ECG and a blood pressure at rest, the patient was placed on a bicycle with an airtight mask for measurements of O_2_ and CO_2_ concentrations. ECG, blood pressure ,and oxygen saturation were also recorded during exercise. The starting workload in Watts and the increase of W per minute (ramp) were adjusted for each patient according to gender, age, and general fitness. The same ramp program was chosen at baseline and follow-up for each patient. VO_2_ peak in mL·min^−1^ was determined, at peak workload, from the cardiopulmonary exercise test examination.

### Statistical Analysis

Results are presented as median [interquartile range (IQR)]. The software Graph Pad Prism version 6.07 (Graph Pad Software, Inc., La Jolla, CA, USA) was used for all statistical analyses. A Wilcoxon matched-pairs signed rank test was used for assessment of differences between baseline and follow-up for VO_2_ peak, global LVEF, regional wall thickening, and global and regional MFR. A Mann-Whitney test was used for assessment of difference in regional wall thickening in ischemic and non-ischemic territories at baseline. A binary logistic regression was built for predicting the improvement in regional myocardial flow reserve. The potential predictors that were considered in the model include baseline coronary flow reserve less than 2.0, revascularization, presence of infarction, baseline rate pressure product, hyperemic rate pressure product, and medication with beta blockers. A *P* value < 0.05 was considered to indicate statistical significance.

## Results

Patient characteristics are shown in Table 1. A total of 19/33 patients (27/99 vessels) were revascularized, 16 by PCI and 3 by CABG. No patient underwent additional PCIs between baseline and follow-up. At baseline, infarct by LGE was found in 13/33 patients (6 LAD, 7 RCA, and 5 LCX) with a scar size of 10 ± 6%. Septal fibrosis of non-ischemic origin was detected in one patient. Following CABG, one patient had an infarct by LGE CMR (scar size: 6%) not seen at baseline. The binary logistic regression model built for predicting improvement in MFR showed significance for the variables “revascularization” (*P *= 0.04) and “baseline MFR less than 2.0” (*P *< 0.001). No statistical significance was found for other potential predictors in the model including the presence of infarction (15/99, *P *= 0.4), rest rate pressure product (9882 ± 1511, *P* = 0.7), hyperemic rate pressure product (10887 ± 2309, *P *= 0.1) ,and medication with beta blockers (48/99, *P* = 0.1).

**Table 1:** Baseline characteristics of the included 33 patients

	Revascularized	Non-revascularized	Total population
Number of patients	19	14	33
Females	5 (26%)	5 (36%)	10 (30%)
Age, years	68 ± 7	67 ± 7	68 ± 7
Baseline examinations to CA, weeks	4 ± 3	5 ± 4	4.5 ± 4
CA to follow-up examinations, months	5 ± 1	5 ± 1	5 ± 1
BMI, kg·m^−2^	28 ± 4	28 ± 4	28 ± 4
Smoker	2 (11%)	1 (7%)	3 (9%)
Previous smoker	10 (53%)	8 (57%)	18 (55%)
Prior PCI	9 (47%)	6 (43%)	15 (45%)
Prior CABG	2 (11%)	0 (0%)	2 (6%)
Prior myocardial infarction	6 (32%)	3 (21%)	9 (27%)
Diabetes	3 (16%)	3 (21%)	6 (18%)
Hypertension	12 (63%)	10 (71%)	22 (67%)
Hypercholesterolemia	13 (68%)	8 (57%)	21 (64%)
Heredity for coronary artery disease	5 (26%)	4 (29%)	9 (27%)
Beta blockers at baseline	9 (47%)	9 (64%)	18 (55%)
Beta blockers during follow-up	14 (74%)	10 (71%)	24 (73%)
ACE-inhibitor/ARB at baseline	10 (53%)	7 (50%)	17 (52%)
ACE-inhibitor/ARB during follow-up	14 (74%)	8 (57%)	22 (67%)
Statins at baseline	18 (95%)	13 (93%)	31 (94%)
Statins during follow-up	19 (100%)	14 (100%)	33 (100%)
Anti-coagulants at baseline	10 (53%)	7 (50%)	17 (52%)
Anti-coagulants during follow-up	17 (89%)	9 (64%)	26 (79%)
Myocardial infarction by LGE	10 (53%)	3 (21%)	13 (39%)

*CA*, coronary angiography; *BMI*, body mass index; *PCI*, percutaneous coronary intervention; *CABG*, coronary artery bypass graft; *ACE*, angiotensin converting enzyme; *ARB*, angiotensin II receptor blockers; *LGE*, late gadolinium enhancement

### Assessment of Myocardial Perfusion Prior to Revascularization

**Figure 1:**
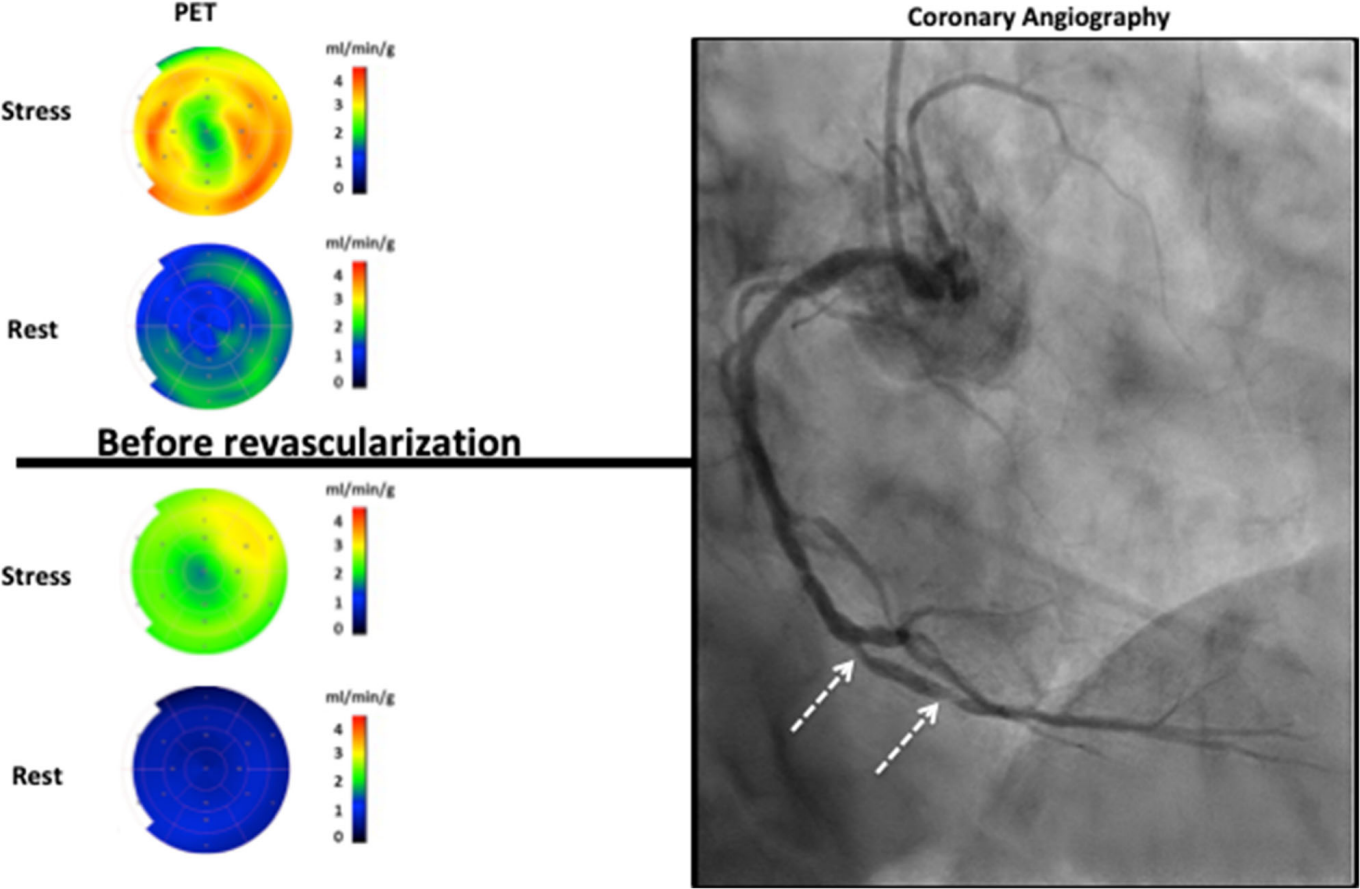
An example of quantitative cardiac positron emission tomography (PET) findings in a patient undergoing revascularization in the RCA based on coronary angiography findings (white arrows). PET bull’s eye plots represent the distribution of the quantified absolute myocardial blood flows (mL·min^−1^·g^−1^ tissue) in the left ventricle. The color scales to the right of the bull’s eyes represent the flow ranges, with yellow red colors indicating higher flows and blue-green colors lower flows. In this case, the patient had a normal regional myocardial flow reserve (MFR) by cardiac PET in the RCA (MFR: 2.7)

In 41% (11/27) of the revascularized vessel territories (4/19 patients), a normal regional MFR was found prior to the PCI (Figure 1).

During CA, invasive flow reserve measurements were performed in 6/19 patients (*n* = 9 vessels, iFR = 8 vessels and FFR = 1 vessel) in the revascularized group and 7/14 patients (*n* = 7 vessels, iFR = 5 vessels and FFR = 2 vessels) in the non-revascularized group. Disagreement between findings of iFR/FFR during CA and MFR by PET was found in one non-revascularized vessel territory with a normal iFR (0.95), but a regional MFR of 1.9 at baseline by PET. In the rest of the cases (*n* = 12 patients, *n* = 15 vessels), there was a complete agreement between iFR/FFR and MFR by PET in discriminating normal and abnormal flow reserve.

An abnormal PET, in one or more coronary territories, was found in 4/14 patients (8/42 vessel territories) not undergoing PCI or CABG. Of those four patients, two had diabetes, one had dilated cardiomyopathy, and one had untreated hypertension. Of note, these patients did not undergo iFR/FFR.

### Effect of Revascularization on MFR and Absolute Myocardial Perfusion

**Figure 2:**
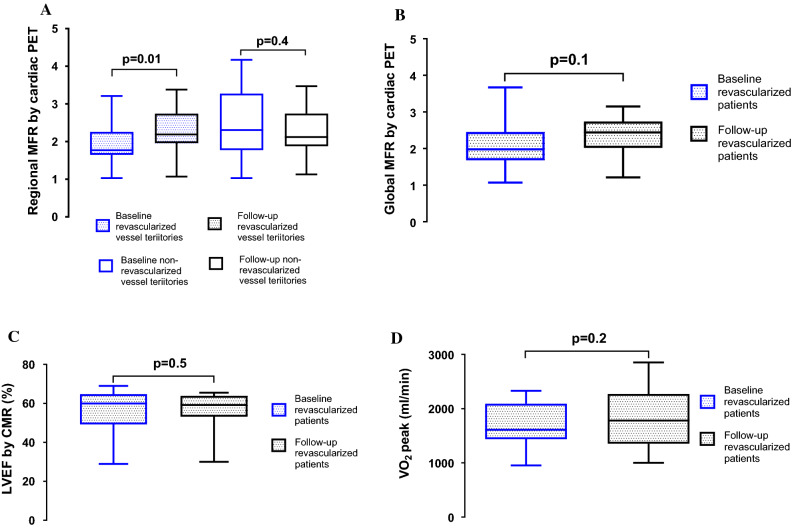
Box and whisker’s plots of the (**A**) regional myocardial flow reserve (MFR) in 27 revascularized and 72 non-revascularized vessel territories, (**B**) global MFR, (**C**) global left ventricular ejection fraction (LVEF), and (**D**) peak oxygen uptake (VO_2_ peak) in patients before (baseline) and after (follow-up) revascularization

**Figure 3:**
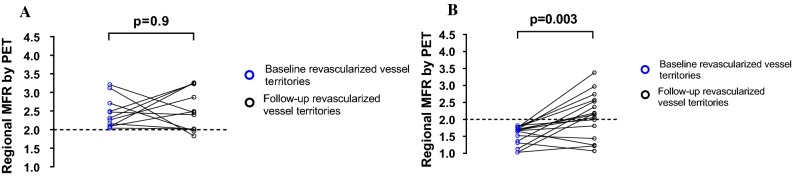
Effect of revascularization (follow-up) on myocardial flow reserve (MFR), as assessed by PET, in vessel territories with a baseline regional MFR (**A**) >2.0 (*n *= 11) and (**B**) <2.0 (*n *= 16). Note that a significant improvement in MFR for the vessel territories with decreased MFR prior to revascularization is not seen in vessel territories with normal MFR at baseline. The dashed line represents the cut-off value of regional MFR at 2.0

In the revascularized patients, no significant change in global MFR was found between baseline and follow-up (2.1 [1.1 to 3.7] vs 2.3[1.2 to 3.2], *P *= 0.1, Figure 2B), whereas for regional MFR, the improvement was significant (1.9 [1.0 to 3.2] vs 2.3 [1.0 to 3.3], *P *= 0.01, Figure 2A) despite the lack of improvement in 9 out of 27 vessel territories. In 41% (11/27) of the revascularized vessel territories, a normal regional MFR (MFR > 2) was seen prior to the PCI, and no improvement in MFR (2.5 [2.0 to 3.2] vs 2.5 [1.8 to 3.3], *P *= 0.9; Figure 3A) and absolute regional myocardial perfusion at stress (2.0 mL·min^−1^·g^−1^ [1.3 to 2.2 mL·min^−1^·g^−1^] vs 2.0 mL·min^−1^·g^−1^ [1.2 to 3.0 mL·min^−1^·g^−1^], *P *= 0.2) was seen at follow-up.

For vessel territories with regional MFR < 2.0 at baseline (*n* = 16 vessels), however, a significant improvement in MFR (1.5 [1.03 to 1.8] vs 2.1 [1.07 to 3.4], *P *= 0.003; Figure 3B) and absolute regional myocardial perfusion at stress (1.5 mL·min^−1^·g^−1^ [0.9 to 2.4 mL·min^−1^·g^−1^] vs 1.9 mL·min^−1^·g^−1^ [1.0 to 2.7 mL·min^−1^·g^−1^], *P *= 0.006) was found when PCI was performed in that vessel (11/16 vessels). In 5/19 revascularized patients, a decrease in regional MFR was seen in the non-revascularized remote myocardium. A baseline sub-endocardial infarct by LGE CMR was found in 8/27 revascularized myocardial territories. An improvement in MFR after revascularization was found in 5/8 revascularized territories which corresponded to a myocardial infarct by LGE.

For the non-revascularized patients, no significant change was found between baseline and follow-up in regional (2.6 [1.9 to 3.1] vs 2.8 [2.0 to 3.0], *P *= 0.6) or global MFR (2.5 [2.1 to 3.1] vs 2.8 [2.1 to 3.2], *P *= 0.9). This included three patients (6 vessels) with MFR < 2.0 at baseline who were not treated with PCI or CABG and had remaining MFR < 2.0 at follow-up.

### Effect of Revascularization on Myocardial Function and Exercise Capacity

No significant change was found between baseline and follow-up with regard to LVEF on CMR or VO_2_ peak in the revascularized patients (LVEF: 60% [49% to 65%] vs 59% [53% to 64%], *P *= 0.5; VO_2_ peak: 1612 mL·min^−1^ [1437 to 2090 mL·min^−1^] vs 1780 mL·min^−1^ [1355 to 2270 mL·min^−1^], *P *= 0.2; Figure 1). The same was found for the non-revascularized patients (LVEF: 62% [54% to 63%] vs 59% [52% to 66%], *P *= 0.9; VO_2_ peak: 1630 mL·min^−1^ [1480 to 2483 mL·min^−1^] vs 1730 mL·min^−1^ [1445 to 2550 mL·min^−1^], *P *= 0.5).

In revascularized patients, there was a significant difference in regional wall thickening on CMR between ischemic (MFR < 2.0) and non-ischemic myocardium (MFR > 2.0) at baseline (38% [24% to 60%] and 89% [66% to 142%], *P *< 0.0001). There was, however, no significant change in regional wall thickening from baseline to follow-up in the myocardial territory supplied by revascularized vessels (42% [24% to 98%] vs 60% [32% to 72%], *P *= 0.9). Of note, some of the revascularized vessels had MFR > 2.0 at baseline (Figure 2A)

## Discussion

The present study shows that assessment of quantitative myocardial perfusion prior to revascularization could lead to more appropriate use of CA when managing patients with stable CAD. In the present study, the angiographer was blinded to the baseline PET and CMR results, and therefore the decision to perform a revascularization was based on clinical data and CA findings only. Not knowing the baseline PET perfusion results led to the revascularization of nine patients without any evidence of ischemia in the territory at baseline and no benefit in perfusion at follow-up, and three patients not being revascularized despite a pathologically decreased perfusion at baseline that remained at follow-up. Increased use of quantitative non-invasive perfusion assessment before revascularization or iFR/FFR during routine CA is warranted.

### Impact of Non-invasive Quantitative Assessment of Perfusion

In the present study, more than 1/3 of the invasive CAs could potentially be avoided, if the revascularization decision was guided by non-invasive assessment of quantitative myocardial perfusion prior to the procedure as suggested by current guidelines.^1^,^24^,^25^ This is supported by the recently published CE-MARC 2 study showing that the number of normal angiographies was significantly lower using a non-invasive imaging strategy (CMR or myocardial perfusion SPECT) compared to the use of the NICE guidelines which recommends CA without prior stress testing of the pre-test likelihood > 85%.^14^ By decreasing the number of unnecessary angiographies, the patients can be spared from the possible complications of the invasive procedure (i.e., endothelial damage and subsequent in-stent stenosis) with no negative effect on major adverse cardiac events.^14^,^15^ Also, invasive procedures are shown to be associated with treatment-related risks such as bleeding and induction of myocardial infarction,^1^ as was found for one of the patients in the present study, and higher health care costs.

For the revascularized patients, potential unnecessary CA-guided revascularizations could have been avoided in 33% (9/27) of the vessel territories if findings of the presence of myocardial perfusion by non-invasive cardiac imaging would have been known by the clinicians prior to the intervention. This supports the previous literature showing that qualitative CA has lower diagnostic accuracy for assessment of stress-induced myocardial ischemia compared to functional imaging methods.^26^

### Effect of Revascularization on MFR

In 5/19 of the revascularized patients, the increase in regional MFR in the revascularized territories was associated with a decrease in regional MFR in the non-revascularized remote territories, as found in the present study. This might be because other factors such as heart rate, blood pressure, presence of infarct, and medication might have influenced the change in MFR from baseline to follow-up in these patients.

### Effect of Revascularization on Myocardial Function

In the present study, revascularization did not improve LVEF in the revascularized patients, which supports previous findings showing that LVEF was unchanged at one month and six months following revascularization.^27^ This might be explained by a combination of increase in regional function in ischemic myocardium and a decrease in regional function in remote myocardium after revascularization, resulting in no significant change in global function.

### Limitations

When interpreting the results from this study, some limitations should be considered. First, the patients included in the present study were recruited from those waiting for an elective CA. Thus, these patients have a higher pre-test likelihood than chest pain patients in general and the conclusions cannot be generalized for the latter. Second, the use of CMR scanners from two different vendors might affect the CMR measurements. Third, the majority of the revascularized patients had significant stenosis in the LAD (63%) and most of these patients (74%) had single vessel disease by CA, which makes the generalization of the findings in the study somewhat limited for all coronary vessels and patients with multi-vessel disease. Fourth, comorbidities, such as dilated cardiomyopathy and microvascular disease, resulting from risk factors such as diabetes or hypertension, can also cause decreased MFR in the absence of significant epicardial CAD. This might explain the normal angiogram in four patients with abnormal MFR by PET and clinical history of diabetes, hypertension, and dilated cardiomyopathy. In this situation, the decision not to revascularize would be considered accurate, despite a low MFR within the vessel territory of interest. Finally, the small population size included in this study is a limitation.

## New Knowledge Gained

The current study shows that patients revascularized based on angiographic and clinical findings do not benefit from the intervention if they have a normal MFR as quantified by PET before intervention. However, the myocardial perfusion of patients with abnormal MFR prior to intervention improves significantly after PCI. This study thus highlights the benefit of quantification with cardiac PET prior to treatment decision.

## Conclusion

Assessment of quantitative myocardial perfusion prior to revascularization could lead to more appropriate use of CA when managing patients with stable CAD. Therefore, unnecessary angiographies and subsequent revascularizations can be avoided.

## Electronic supplementary material

Below is the link to the electronic supplementary material.

Supplementary material 1 (PPTX 156 kb)

Supplementary material 2 (XSPF 156 kb)

## Abbreviations

PCI: Percutaneous coronary intervention
CAD: Coronary artery disease
MFR: Myocardial flow reserve
CA: Coronary angiography
CMR: Cardiac magnetic resonance imaging
LGE: Late gadolinium enhancement
LVEF: Left ventricular ejection fraction
