# Supplementary material for: Appropriate coronary revascularization can be accomplished if myocardial perfusion is quantified by positron emission tomography prior to treatment decision
Source: J Nucl Cardiol. 2019 Nov 8;28(4):1664–72. doi: 10.1007/s12350-019-01938-y (PMC8421314; doi:10.1007/s12350-019-01938-y)
Supplement: Supplementary file 1 — Supplementary material 1 (PPTX 156 kb) [file 12350_2019_1938_MOESM1_ESM.pptx]

## Slide 1
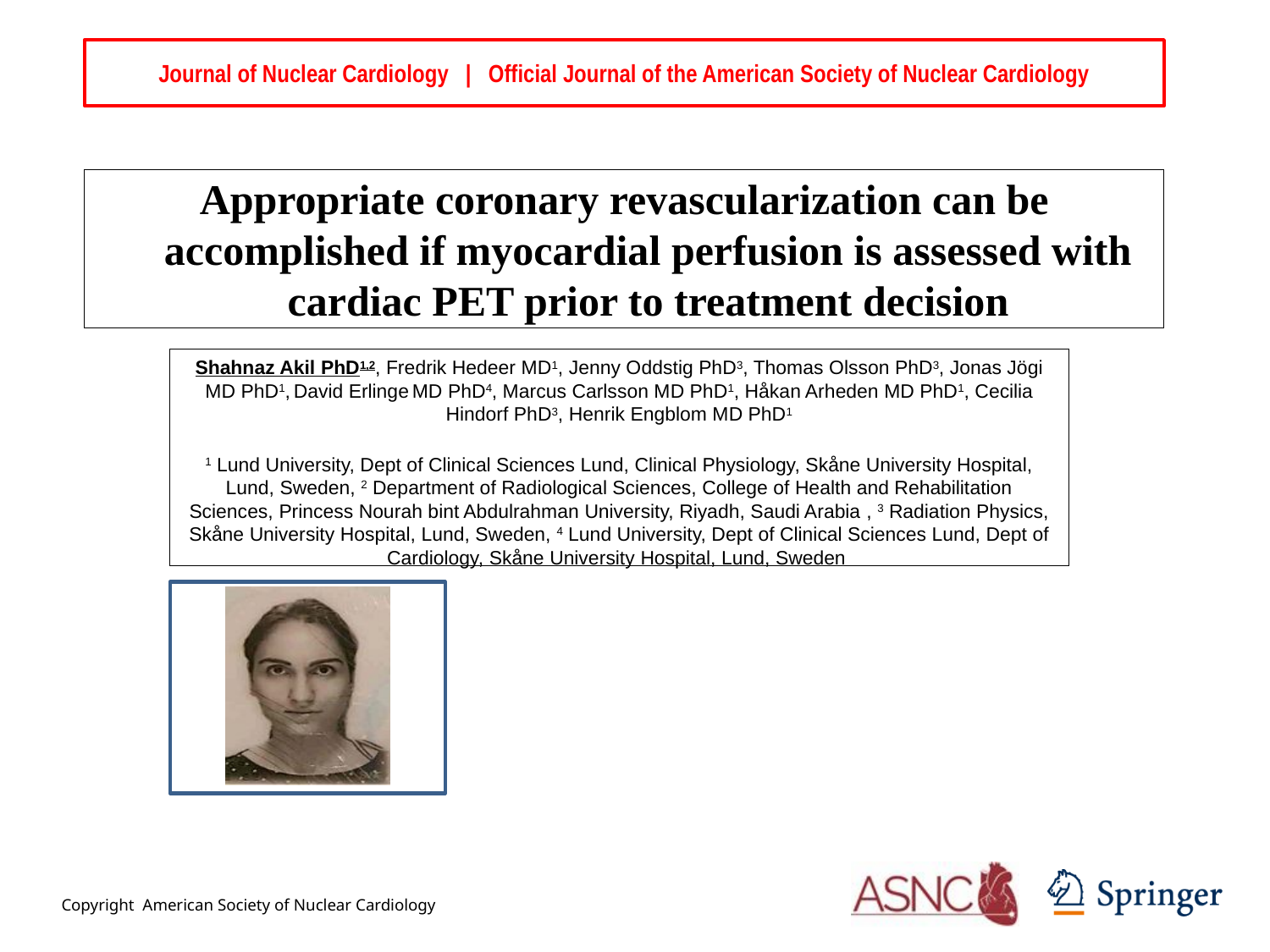

Journal of Nuclear Cardiology | Official Journal of the American Society of Nuclear Cardiology
# Appropriate coronary revascularization can be accomplished if myocardial perfusion is assessed with cardiac PET prior to treatment decision
Shahnaz Akil PhD1,2, Fredrik Hedeer MD1, Jenny Oddstig PhD3, Thomas Olsson PhD3, Jonas Jögi MD PhD1, David Erlinge MD PhD4, Marcus Carlsson MD PhD1, Håkan Arheden MD PhD1, Cecilia Hindorf PhD3, Henrik Engblom MD PhD1
1 Lund University, Dept of Clinical Sciences Lund, Clinical Physiology, Skåne University Hospital, Lund, Sweden, 2 Department of Radiological Sciences, College of Health and Rehabilitation Sciences, Princess Nourah bint Abdulrahman University, Riyadh, Saudi Arabia , 3 Radiation Physics, Skåne University Hospital, Lund, Sweden, 4 Lund University, Dept of Clinical Sciences Lund, Dept of Cardiology, Skåne University Hospital, Lund, Sweden
Head
Copyright American Society of Nuclear Cardiology

## Slide 2
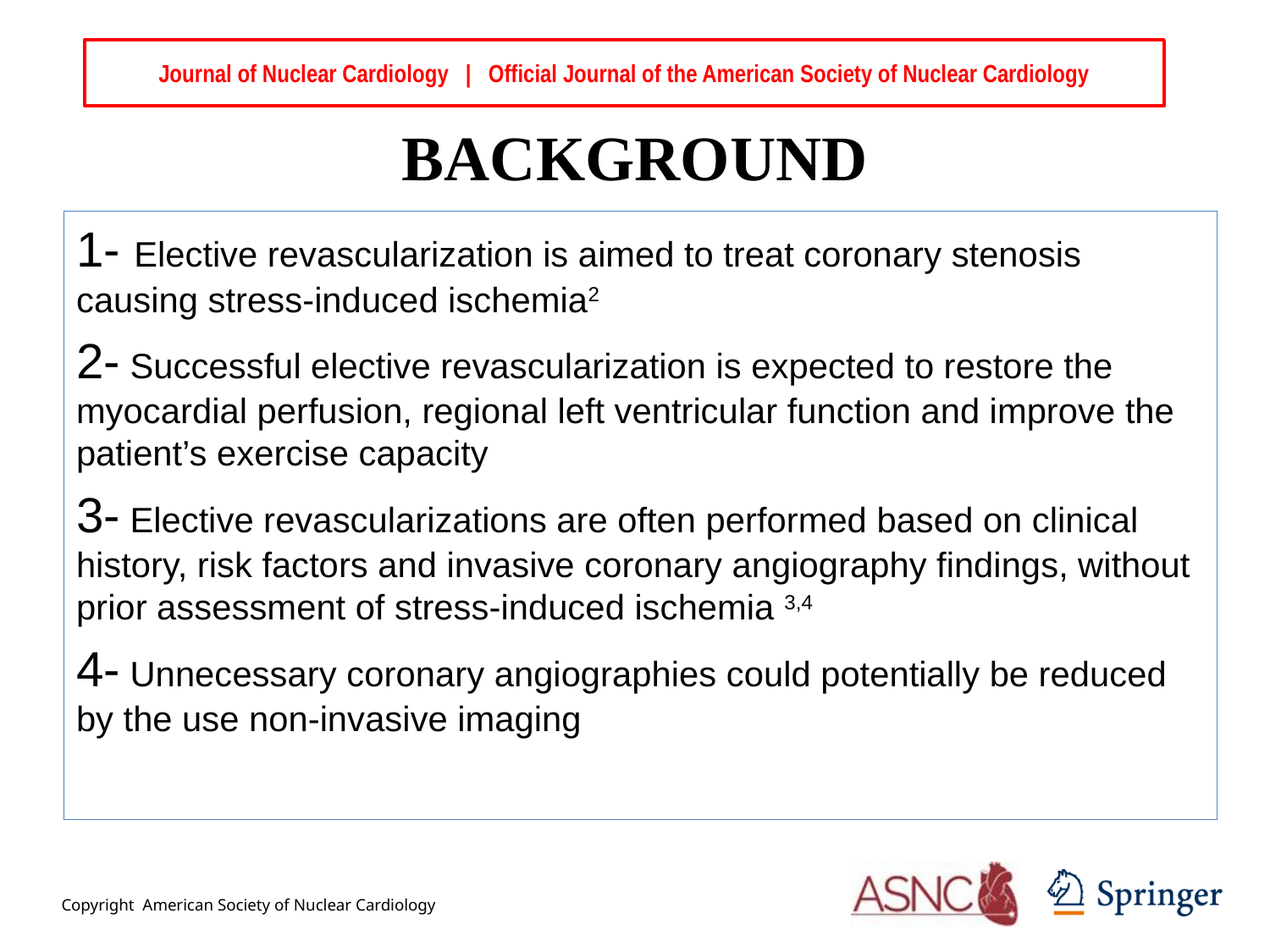

Journal of Nuclear Cardiology | Official Journal of the American Society of Nuclear Cardiology
# BACKGROUND
1- Elective revascularization is aimed to treat coronary stenosis causing stress-induced ischemia2
2- Successful elective revascularization is expected to restore the myocardial perfusion, regional left ventricular function and improve the patient’s exercise capacity
3- Elective revascularizations are often performed based on clinical history, risk factors and invasive coronary angiography findings, without prior assessment of stress-induced ischemia 3,4
4- Unnecessary coronary angiographies could potentially be reduced by the use non-invasive imaging
Copyright American Society of Nuclear Cardiology

## Slide 3
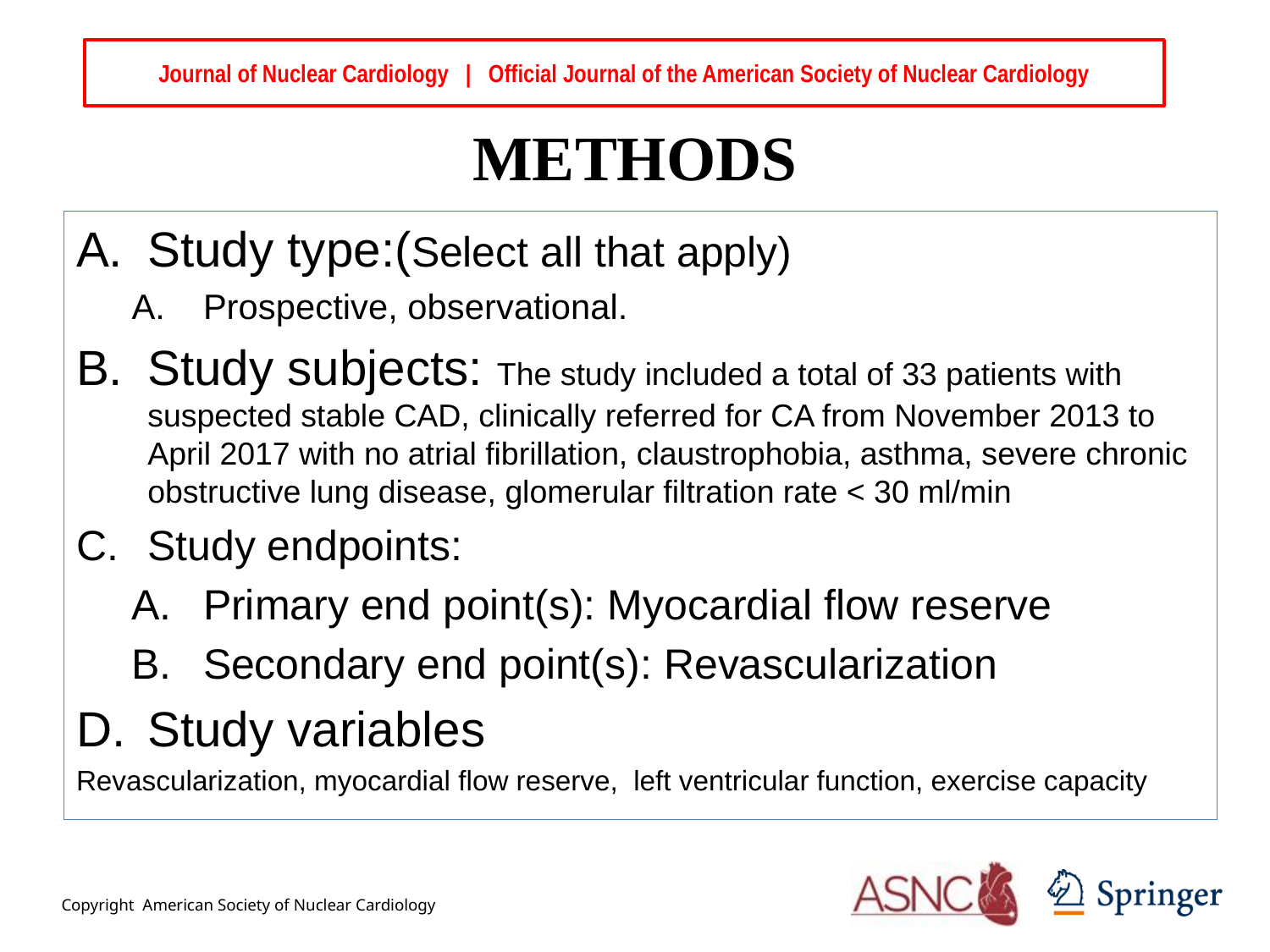

Journal of Nuclear Cardiology | Official Journal of the American Society of Nuclear Cardiology
# METHODS
Study type:(Select all that apply)
Prospective, observational.
Study subjects: The study included a total of 33 patients with suspected stable CAD, clinically referred for CA from November 2013 to April 2017 with no atrial fibrillation, claustrophobia, asthma, severe chronic obstructive lung disease, glomerular filtration rate < 30 ml/min
Study endpoints:
Primary end point(s): Myocardial flow reserve
Secondary end point(s): Revascularization
Study variables
Revascularization, myocardial flow reserve, left ventricular function, exercise capacity
Copyright American Society of Nuclear Cardiology

## Slide 4
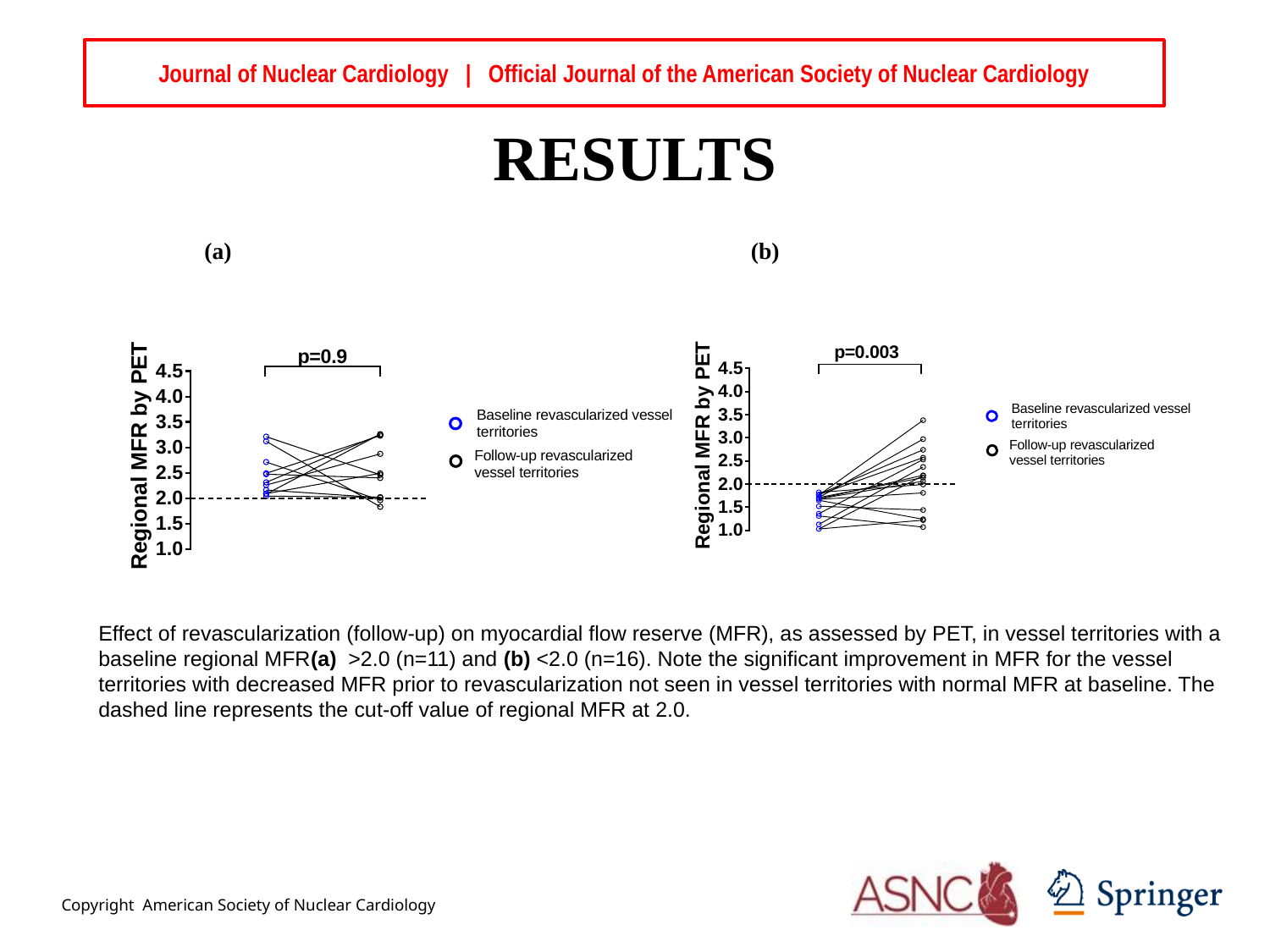

Journal of Nuclear Cardiology | Official Journal of the American Society of Nuclear Cardiology
# RESULTS
Effect of revascularization (follow-up) on myocardial flow reserve (MFR), as assessed by PET, in vessel territories with a baseline regional MFR(a) >2.0 (n=11) and (b) <2.0 (n=16). Note the significant improvement in MFR for the vessel territories with decreased MFR prior to revascularization not seen in vessel territories with normal MFR at baseline. The dashed line represents the cut-off value of regional MFR at 2.0.
Copyright American Society of Nuclear Cardiology

## Slide 5
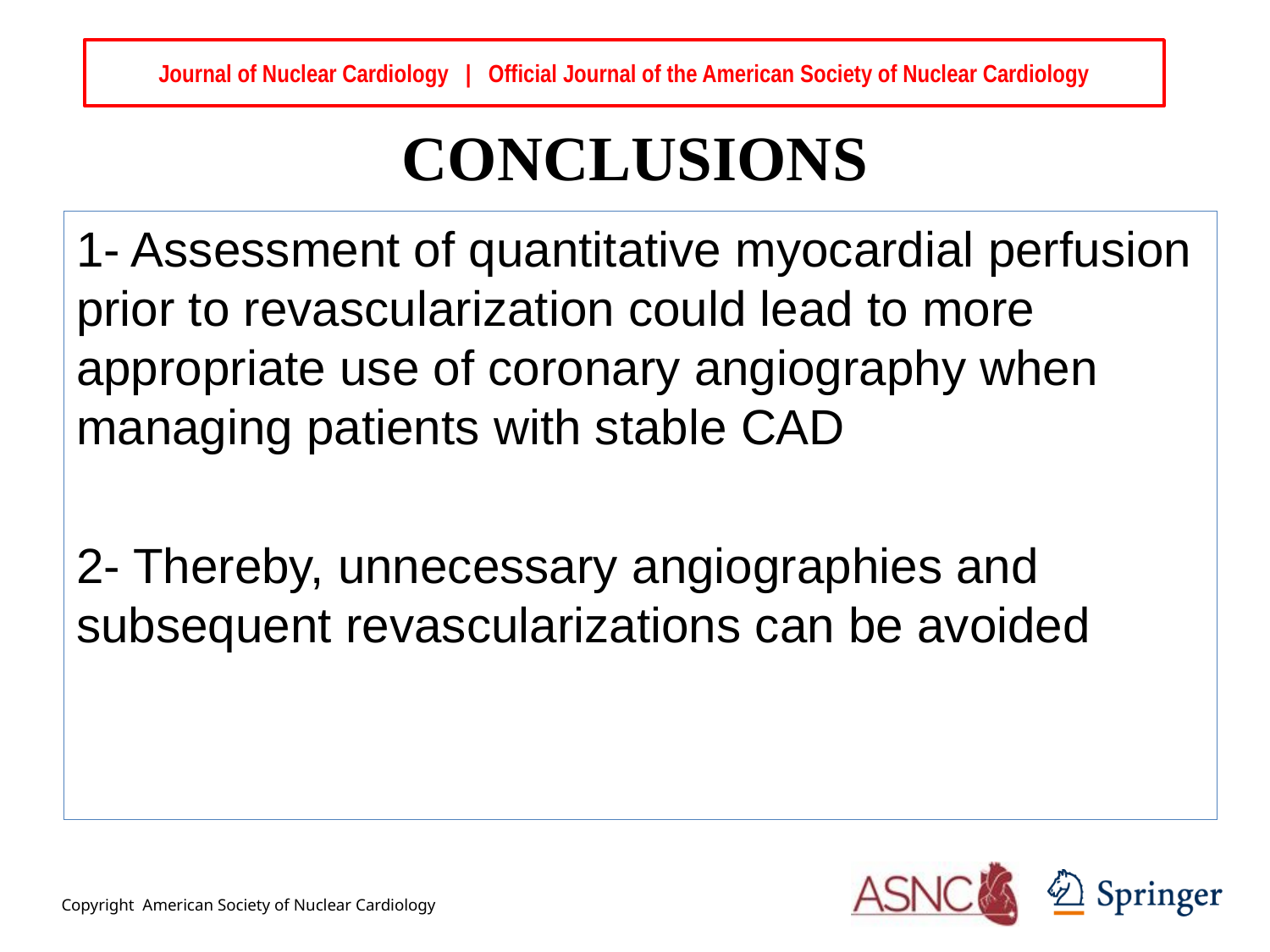

Journal of Nuclear Cardiology | Official Journal of the American Society of Nuclear Cardiology
# CONCLUSIONS
1- Assessment of quantitative myocardial perfusion prior to revascularization could lead to more appropriate use of coronary angiography when managing patients with stable CAD
2- Thereby, unnecessary angiographies and subsequent revascularizations can be avoided
Copyright American Society of Nuclear Cardiology
